# Gender Differences Amongst First-Time Admitted Psychiatric Inpatients in Palestine

**DOI:** 10.1192/bjo.2024.203

**Published:** 2024-08-01

**Authors:** Nizar Marzouqa, Thaer Alhroob, Wala Alnammourah, Asala Awwad, Layth Oweina

**Affiliations:** Bethlehem Psychiatric Hospital, Bethlehem, Palestine

## Abstract

**Aims:**

Bethlehem Psychiatric Hospital is the only psychiatric hospital in the West Bank. Gender differences aren't widely studied in medical Palestinian research, therefore, data on it is very scarce. This study aims to study gender-based patterns of clinical and demographic characteristics amongst patients admitted for the first time at Bethlehem Psychiatric Hospital over a year.

**Methods:**

A retrospective cross-sectional study was conducted at the Bethlehem Psychiatric Hospital, reviewing the medical records of patients admitted for the first time between October 2022 and October 2023. First, data collection was conducted manually by residents transferring information from paper-based files to an Excel sheet. Next, sociodemographic and clinical variables were selected. Finally, the 21st version of IBM SPSS was used to analyze the role of gender factors.

**Results:**

For the 140 patients admitted for the first time to the psychiatric hospital between October 2022 and October 2023, the majority (70%) were male, with a mean age of 31.6 for males and 35 for females.

Most variables showed no significant differences between male and female patients. Of the variables that showed significantly (p < 0.05) higher occurrence in male patients were imprisonment, physical aggression (78.6% of males, 61.9% of females), smoking (84.4% of males, 14.3% of females), and substance use (36.7% of males, 2.3% of females). However, length of stay, clozapine prescription, and parents' consanguinity were significantly higher in women.

**Conclusion:**

This is the first study conducted about gender differences in Palestinian psychiatric inpatients. Some elements pointing to antisocial traits (like imprisonment and substance use) were more common in males, while women stayed longer at the hospital. Studying these measures and their etiology is crucial for better understanding and management.

There is a need for more research on gender differences, and Palestinian psychiatry as a whole, integrating social-economic, cultural, and medical views, to provide better equitable care for patients, and be able to advocate better for them.

